# BRCA1 mutations in high-grade serous ovarian cancer are associated with proteomic changes in DNA repair, splicing, transcription regulation and signaling

**DOI:** 10.1038/s41598-022-08461-0

**Published:** 2022-03-15

**Authors:** Melissa Bradbury, Eva Borràs, Josep Castellví, Olga Méndez, José Luis Sánchez-Iglesias, Assumpció Pérez-Benavente, Antonio Gil-Moreno, Eduard Sabidó, Anna Santamaria

**Affiliations:** 1 Proteomics Unit, Centre de Regulació Genòmica, Barcelona Institute of Science and Technology (BIST), Dr Aiguader 88, 08003 Barcelona, Spain; 2 Universitat Pompeu Fabra, Dr Aiguader 88, 08003 Barcelona, Spain; 3 Biomedical Research Group in Gynecology, Vall d’Hebron Institut de Recerca, Vall d’Hebron Barcelona Hospital Campus, Universitat Autònoma de Barcelona, Passeig Vall d’Hebron 119-129, 08035 Barcelona, Spain; 4 Department of Gynecology, Hospital Universitari Vall d’Hebron, Vall d’Hebron Barcelona Hospital Campus, Passeig Vall d’Hebron 119-129, 08035 Barcelona, Spain; 5 Department of Pathology, Hospital Universitari Vall d’Hebron, Vall d’Hebron Barcelona Hospital Campus, Passeig Vall d’Hebron 119-129, 08035 Barcelona, Spain; 6 Centro de Investigación Biomédica en Red (CIBERONC), Instituto de Salud Carlos III, Avenida de Monforte de Lemos 3-5, 28029 Madrid, Spain; 7 Present Address: Cell Cycle and Cancer Laboratory, Biomedical Research Group in Urology, Vall Hebron Institut de Recerca, Vall d’Hebron Barcelona Hospital Campus, Universitat Autònoma de Barcelona, Passeig Vall d’Hebron 119-129, 08035 Barcelona, Spain

**Keywords:** Proteomics, Ovarian cancer

## Abstract

Despite recent advances in the management of BRCA1 mutated high-grade serous ovarian cancer (HGSC), the physiology of these tumors remains poorly understood. Here we provide a comprehensive molecular understanding of the signaling processes that drive HGSC pathogenesis with the addition of valuable ubiquitination profiling, and their dependency on BRCA1 mutation-state directly in patient-derived tissues. Using a multilayered proteomic approach, we show the tight coordination between the ubiquitination and phosphorylation regulatory layers and their role in key cellular processes related to BRCA1-dependent HGSC pathogenesis. In addition, we identify key bridging proteins, kinase activity, and post-translational modifications responsible for molding distinct cancer phenotypes, thus providing new opportunities for therapeutic intervention, and ultimately advance towards a more personalized patient care.

## Introduction

Germline mutations in the BRCA1 gene are the most common hereditary factor associated with the development of high-grade serous ovarian cancer (HGSC)^1–3^. In current clinical practice these mutations provide a rationale for treatment with poly(ADP-ribose) polymerase (PARP) inhibitors in the maintenance and recurrent setting and the implementation of close surveillance and risk-reducing strategies^1,4^. BRCA1 plays a primarily role in the DNA damage response and the regulation of transcription^5–7^. BRCA1 has two highly conserved domains: a N-terminal RING domain, which has a E3 ubiquitin ligase activity through the interaction with BRCA1-associated RING domain 1 (BARD1)^6^, and a tandem BRCT domain at its C-terminus, that mediates phosphorylation-dependent interactions with other proteins^5^. Despite the advances made in vitro to decipher BRCA1 cellular functions, the importance of BRCA1 mutations in the clinical setting and the rapid escalation of the clinical use of PARP inhibitors, a deeper understanding of HGSC tumor molecular phenotypes is still necessary. This would allow the identification of new therapeutic targets in order to advance towards a more streamlined and personalized care.

Mass spectrometry-based proteomics enables large-scale analyses of protein expression and their post-translational modifications (PTM)^8^. Although many studies have analyzed protein changes in ovarian cancer cell lines for the investigation of signaling events, cellular perturbations and response to therapies, few have analyzed them directly in ovarian cancer tissues^9–12^. These studies rely on the characterization of the proteome and phosphorylation changes, with none having integrated changes occurring at the level of ubiquitination. Indeed, little is known on how the ubiquitinated state of particular proteins vary in ovarian cancer tissues and their association to highly prevalent mutations such as BRCA1. Furthermore, phosphorylation and ubiquitination can interplay through a variety of mechanisms including the phosphorylation-mediated regulation of the activity of E3 ligases or the ubiquitination regulation of kinase activity^13,14^. Despite the growing interest in PTM crosstalk this cellular event remains largely unexplored in human tissues. Here, we demonstrate the coordination between the different regulatory layers in the modulation of key cellular processes in HGSC and its BRCA1-dependent signaling events. We also highlight the importance of BRCA1 substrates in the modulation of HGSC ubiquitination events in vivo and the cellular compartmentalization of BRCA1-dependent signaling processes associated with pathogenic mutations.

## Results

### Phosphoproteomic regulation of key cellular processes is decoupled from proteome changes in HGSC patient-derived tissues

**Figure 1:**
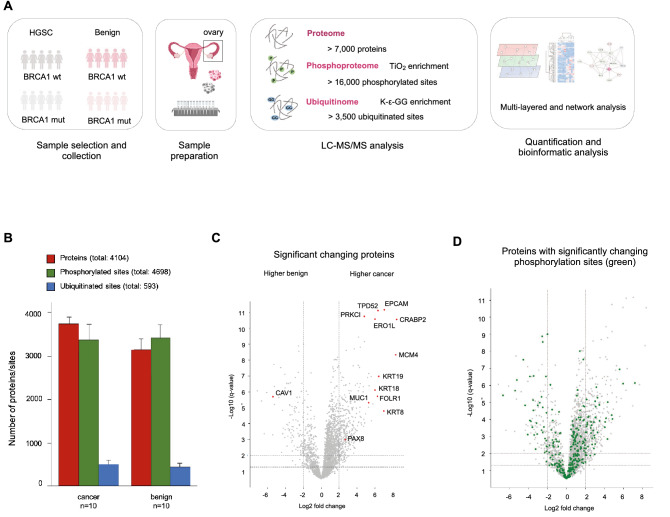
Proteome and phosphoproteome analysis shows differentially regulated signaling processes in ovarian cancer and benign tissues. **(A)** Workflow of the sample preparation and multi-layered proteomic analysis of high-grade serous cancer (HGSC) and benign epithelial ovarian tissues. **(B)** Number of quantified proteins (red), phosphorylated sites, probability score > 0.75 (green) and ubiquitinated sites, probability score > 0.75 (blue) in high-grade serous cancer (HGSC) and benign tissues. Data are presented as mean ± SD. **(C)** Volcano plot of the pairwise comparison between HGSC and benign ovarian tissue proteomes. Marked in red are proteins reported in the literature as being altered in HGSC or epithelial ovarian cancer^15,94–97^. Dotted lines indicate q-value < 0.01 and < 0.05 and log2 fold change > 2 thresholds. Positive and negative log2 fold changes indicate enrichment in cancer and benign tissues, respectively. **(D)** Proteins as in Figure A (grey) with significant phosphorylation sites (green), showing the decoupling between regulated phosphorylated sites and their corresponding protein. Values expressed as log2 fold change of protein expression.

We first aimed to assess the global changes occurring at the level of the proteome and phosphoproteome between HGSC (n = 10, BRCA1wt and BRCA1mut) and benign ovarian human tissues (n = 10) (Tables S1 and S2, Fig. 1A). We identified over 7,000 proteins and 16,000 phosphorylated sites. When compared with previous studies in ovarian cancer, such as the Clinical Proteomic Tumor Analysis Consortium (CPTAC), these numbers were in line with previous observations, showing an overlap of almost 90% of the proteome and 70% of the phosphoproteome^9,10^ (Fig. S1). A total of 4104 proteins and 4698 phosphorylation sites (probability score > 0.75) that were consistently quantified in at least 60% of samples were included in the differential analysis (Fig. 1B). We identified 1,892 differentially expressed proteins between cancer and benign tissues. Some of the most significantly regulated proteins were either known to be markers of HGSC or have been previously described to be altered in epithelial ovarian cancer (EOC) cells^15,16^ (Fig. 1C). Phosphoproteomic analysis revealed 1221 differentially expressed phosphorylated sites encompassing over 709 phosphorylated proteins. Noteworthy, we found a decoupled regulation in HGSC tissues between the phosphorylated peptides and their corresponding protein, i.e. significantly regulated proteins observed at the proteome level did not match with those proteins significantly regulated at the phosphorylation level (Fig. 1D). This observation suggests that both layers of regulation coexist in modulating HGSC tissues and emphasizes the importance of adding the information derived from the phosphoproteome to the proteome dataset to elucidate cellular signaling events. Gene ontology (GO) enrichment analysis revealed that while protein abundances were specifically regulated in cellular processes involved in energy metabolism and translation (Fig. S2), phosphoproteome regulation mainly affected RNA splicing, cell cycle, DNA damage response, nucleosome components and proteins involved in cytoskeleton and cell junction organization. These results agree with those previously observed in tissue-derived ovarian cells, thus confirming the importance of these cellular processes in HGSC pathogenesis, not only in primary cell lines as observed by other authors, but also directly in patient tissue samples^10^.

### Differential ubiquitin linkages in HGSC tissues target canonical cancer hallmarks

**Figure 2:**
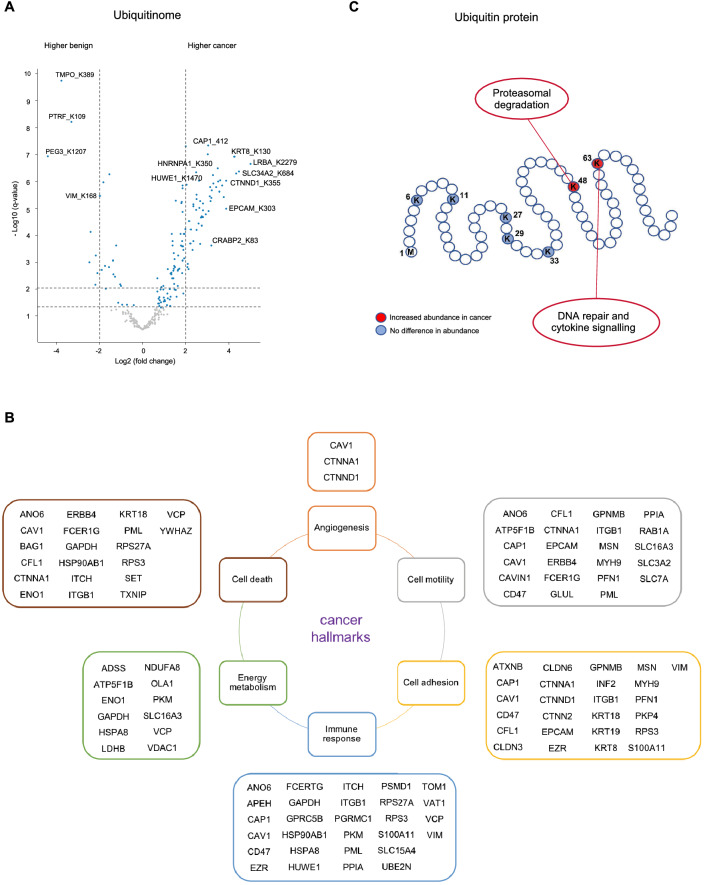
Ubiquitination is involved in the regulation of cancer hallmarks. **(A)** Volcano plot of the pairwise comparison between high-grade serous cancer (HGSC) and benign ovarian tissue ubiquitinomes. Marked in blue are the significantly regulated ubiquitinated sites (q-value < 0.05). These correspond to a total of 151 ubiquitinated sites and 116 proteins. Highlighted are proteins known to be associated with ovarian cancer pathogenesis. **(B)** Cancer hallmarks found regulated by ubiquitination in HGSC and benign tissues. Differentially expressed proteins (q-value < 0.05) belonging to each of the cancer hallmarks are annotated. **(C)** Overview of the ubiquitin lysine (K)-linkage sites identified in our study. Marked in red are the ubiquitinated lysines (K48 and K63) found to be more abundant in HGSC compared to benign tissues.

We next evaluated the changes occurring at the level of the ubiquitinome between HGSC and benign ovarian tissues (Tables S1 and S2, Fig. 1A). No studies to date have evaluated the ubiquitinome in patient-derived fresh frozen tissues, so we provide the first evidence of this approach to study ovarian cancer. We identified 3,500 ubiquitinated sites (probability score > 0.75) (Figure S2). A total of 593 ubiquitination sites (probability score > 0.75) were consistently quantified (Fig. 1B) of which 151, corresponding to 116 proteins, were changing significantly thus confirming the importance of the ubiquitination layer in the regulation of cancer in vivo, in addition to the proteome and phosphoproteome (Fig. 2A). Although some of the proteins identified in our ubiquitination dataset are known to be associated with ovarian cancer, such as CAP1, CRABP2, EPCAM or KRT8 (Fig. 1C), their ubiquitination status has not been previously investigated in HGSC. To explore the role of ubiquitination in this disease, we first checked if any of the proteins showing differential ubiquitination were directly involved in any of these hallmark traits. Most of the proteins regulated at the level of ubiquitination in patient-derived tissues were related to the general hallmarks of cancer^17^ including energy metabolism and cell immunity, two emerging hallmarks which are known to be crucial in ovarian cancer development and progression (Fig. 2B).

Because ubiquitination of the seven lysine residues of ubiquitin are known to have different cellular effects, we next evaluated if ubiquitination of any of these sites changed between HGSC and benign tissues. We identified all seven ubiquitin-linkage sites and found a specific regulation of ubiquitin K48- and K63-linked ubiquitination that has not been previously described in HGSC and links ubiquitination events to the DNA damage response (Fig. 2C). K48 polyubiquitination is associated to protein targeting for proteasomal degradation whilst K63 ubiquitination serves as a scaffold for signaling complex assembly during template switching, double-strand break response and is linked to non-proteolytic functions including cytokine signaling^18,19^. Genomic instability due to defective DNA repair is a common trait in HGSC and is also an important therapeutic target^20^. Noteworthy, several of the regulated ubiquitinated sites identified in our study (e.g. HNRNPC, NEDD8, PCNA, XRCC5) have previously been involved in global DNA damage response changes in vitro^21–23^.

### Crosstalk between the phosphorylation and ubiquitination layers drives HGSC tumorigenesis

**Figure 3:**
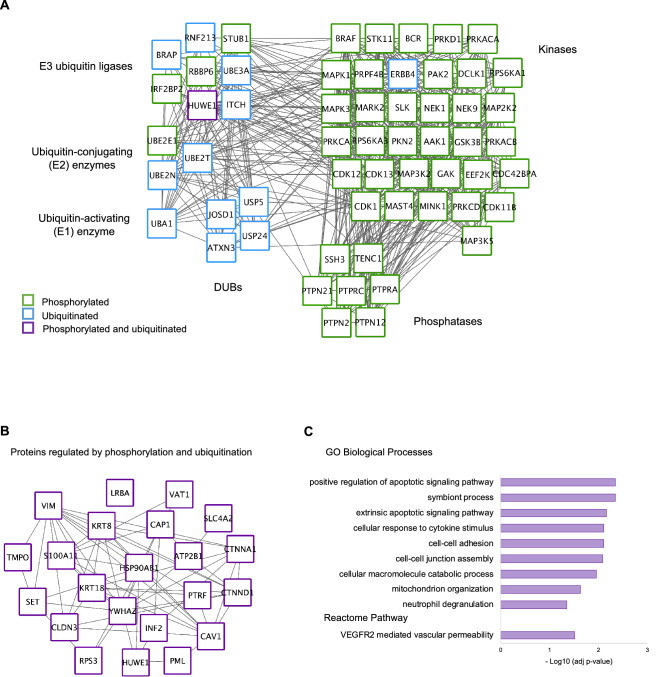
Crosstalk between phosphorylation and ubiquitination in ovarian cancer. **(A)** Network of kinases, phosphatases, ubiquitin-related (E1, E2, E3) and deubiquitinating enzymes (DUBs) modified by phosphorylation (nodes with green border), ubiquitination (blue border) or by both modifications (purple border) between high-grade serous cancer and benign tissues (q-value < 0.05). Edges represent protein–protein associations **(B)** Functional network of the 22 proteins (nodes) regulated by phosphorylation and ubiquitination (q-value < 0.05). Edges represent protein–protein associations **(C)** GOBP and Reactome pathway enrichment analysis of the 22 proteins represented in **(B)**.

The interplay between phosphorylation and ubiquitination in regulating cellular events is well established^13,14^. However, little is known quantitatively on how the writers and erasers of phosphorylation and ubiquitination are altered in the context of specific signaling pathways in human tissues^13^. In fact, no proteomic studies have neither surveyed BRCA1-dependent ubiquitination and phosphorylation by associated kinases together nor explored this crosstalk deeply enough to make meaningful conclusions about reciprocal regulation. To examine such coregulation, we compared the number of post-translationally modified kinases, phosphatases, ubiquitin-related and deubiquitinating enzymes (DUBs). Kinases and phosphatases were mainly regulated by phosphorylation, whereas ubiquitin-related enzymes and DUBs were mainly regulated by ubiquitination (Fig. 3A). However, we did find evidence of a crosstalk between the two systems affecting several ubiquitin-related enzymes. Amongst these was HUWE1, a multifaceted E3 ligase, which has been linked to tumorigenesis and metastasis^24^. HUWE1 is able to catalyze both mono-ubiquitination and K48- and K63-linked polyubiquitination of its substrates to regulate several cellular processes including cell proliferation, apoptosis, immune response and DNA repair^25,26^. Interestingly, in addition to HUWE1, other regulated ubiquitin enzymes found in this study such as ITCH, STUB1, UBA1, UBE2T or UBE2N are also related to DNA repair and K48- and K63-linkage polyubiquitination indicating that both phosphorylation and ubiquitination likely intersect to modulate these cellular events^27,28^.

Because HUWE1 was regulated both at the level of phosphorylation and ubiquitination, we next explored if other proteins were also regulated at the level of both PTMs. Indeed, we identified a total of 22 proteins, in addition to HUWE1, which were regulated both by phosphorylation and ubiquitination corresponding to a total of 82 sites (Fig. 3B and Table S3). GO enrichment and Reactome pathway analysis showed that these 22 proteins were involved in apoptosis, cytokine signaling, cell adhesion and angiogenesis (Fig. 3C). Because site-specific modifications can have unique regulatory effects, we checked if any of the modified sites had been previously investigated regarding their downstream signaling events or their upstream associated kinase or ubiquitin ligase enzymes. For this we searched the PhosphoSitePlus database^29^ and identified 13 known sites corresponding to 8 proteins. Of these, phosphorylation of Vimentin (VIM) at S459 by PRKCD, RPS3 at T221 by PRKDC and ubiquitination of Caveolin-1 (CAV1) at K5 have been linked DNA damage repair, cytoskeleton reorganization and endosomal transport in vitro^30,31^. Other sites such as phosphorylation of CTNND1 at S268 by PKCE or CTNNA1 at T654 have been associated with E-cadherin, Wnt signaling and epithelial-to-mesenchymal transition^32,33^. These modifications have also been associated with tumor progression together with PML ubiquitination and phosphorylation at S402 and S518, and CAP1 phosphorylation by GSK3B at T307, S308 and S310^34–37^. Phosphorylation of HSP90AB1 by CK2 at S226 has been linked to drug resistance^38^. In addition to identifying sites on previously uncharacterized targets (Table S3), we uncovered additional phosphorylation and ubiquitination sites on known players identified in vitro involved in DNA damage and stress response (e.g. CAV1, HSP90AB1, HUWE1, PTRF, YWHAZ)^21,23^. Overall, our results show there is a certain degree of crosstalk between the phosphorylation and ubiquitination layers of regulation in the molecular remodeling in ovarian tissue tumorigenesis*.* Firstly, through the co-regulation of the writers and erasers of each system and secondly, through the co-modification of specific proteins involved in HGSC signaling events. These proteins act as bridges between the two regulatory systems which are key in the tissue physiology of cancer.

### Elucidation of specific BRCA1-dependent regulatory events in HGSC

To determine whether and how BRCA1 mutations influence the proteome, phosphoproteome and ubiquitinome within the cancer patient group we compared the changes in BRCA1mut (n = 5) and BRCA1wt (n = 5) cancer tissues. We first assessed how BRCA1 mutations affected the global proteome. Out of the 4050 quantified proteins we only identified 93 showing differential abundance between BRCA1mut and BRCA1wt tumors and GO term analysis did not show enrichment of any particular pathway (Table S2). The low number of significantly altered protein abundances and the fact that proteins were scattered among different pathways led us to postulate that specific tumor phenotypic differences associated to BRCA1 may not be driven by proteome alterations, but by alterations in protein function through changes in ubiquitination and phosphorylation. Therefore, using our multi-layered dataset we explored, firstly, if changes in ubiquitination between BRCA1mut and BRCA1wt tumors could be related to BRCA1 and, secondly, which kinases were responsible for driving the phosphorylation changes in these tumors and their dependency on BRCA1 mutations.

### BRCA1-dependent ubiquitination landscape in HGSC

**Figure 4:**
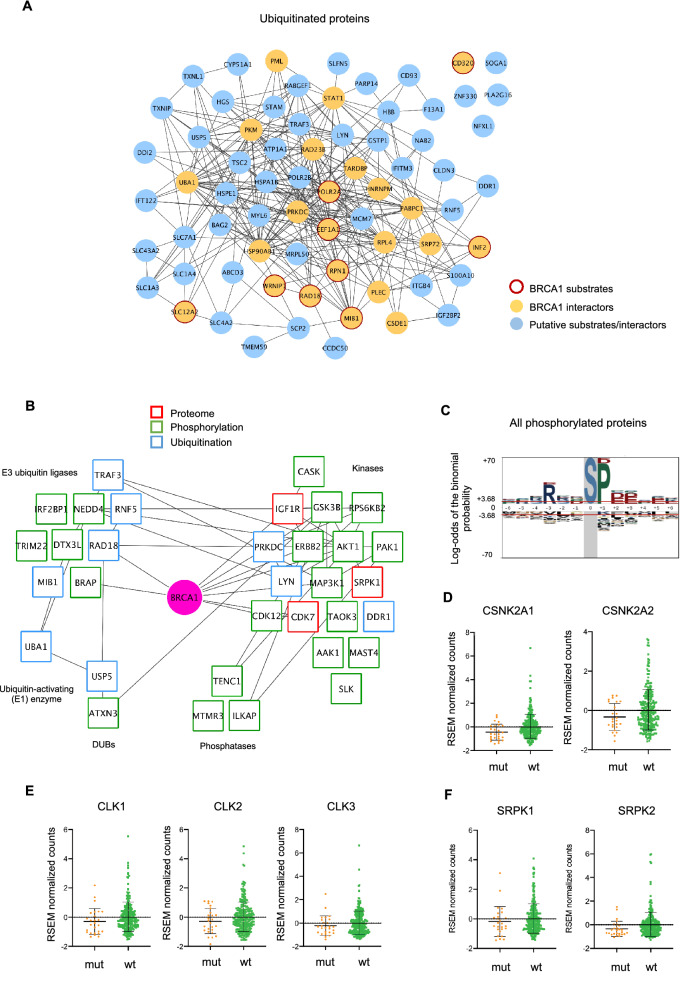
Functional relation between the BRCA1-dependent ubiquitinated proteins identified in our study. **(A)** Network of ubiquitinated proteins regulated in HGSC tissues (q-value < 0.05)**.** Marked in yellow are known BRCA1 interacting proteins and substrates (red border). Marked in blue are proteins which have not been previously associated with BRCA1 and might correspond to novel interactors or substrates. Edges represent protein–protein associations **(B)** Network of the regulated kinases, phosphatases, ubiquitin-related and deubiquitinating (DUB) enzymes (nodes), and their relation with BRCA1 (q-value < 0.05). Colored borders indicate regulation at the level of the proteome (red), phosphorylation (green) or ubiquitination (blue). Data was extracted from STRING and visualized with Cytoscape. **(C)** Sequence motif analysis of the ± six amino acid residues flanking all the regulated phosphorylation sites. The ± 3.68 threshold corresponds to a significant enrichment with p < 0.05. Highlighted in grey is the fixed amino acid position. **(D)** RSEM count (z-score normalized) differences observed between mRNA levels of BRCA1wt and BRCA1mut tumors for CSNK2 subunit A, **(E)** CLK and **(F)** SRPK kinase families. CSNK2A1, p = 0.008; CSNK2A2, p = 0.138; CLK1, p = 0.06; CLK2, p = 0.205; CLK3, p = 0.207; SRPK1, p = 0.292; SRPK2, p = 0.012 (two-sided Mann–Whitney U test). Data obtained from the TCGA ovarian cystadenocarcinoma dataset (Cancer Genome Atlas Research Network, 2011).

We next evaluated the changes occurring at the level of ubiquitination between the cancer tissues with different BRCA1 mutational status. A total of 77 sites corresponding to 70 proteins were found differentially regulated between BRCA1mut and BRCA1wt cancer tissues. We checked if any of these proteins had been previously described to be functionally related to BRCA1^39,40^ and we identified 23 proteins that had been reported in vitro as being substrates or interactors of BRCA1 (Table S4 and Fig. 4A). Despite the innate heterogeneity between tumors, the changes observed in BRCA1 known substrates and interactors suggest that its enzymatic activity is modified in BRCA1 mutated tumors. These proteins are summarized in Table S4. These include, amongst others, POLR2A, PML, EEF1A1, MIB1, PKM, STAT1 and UBA1. In addition to these known substrates and interactors, we also identified a substantial number of other differentially ubiquitinated proteins which constitute potential direct or indirect novel BRCA1 substrates not previously described (Table S4 and Fig. 4A).

Because BRCA1 is known to catalyze K6-, K48-, and K63-linked polyubiquitin chains, we assessed if these sites changed upon BRCA1 mutation in HGSC in the same way as previously performed for tumors and benign epithelial ovarian tissues. Although we were able to identify all seven linkage sites, we did not find any changes in their peptide abundances. These results were in line with those previously reported in non-ovarian cell lines^41^, reinforcing the idea that BRCA1/BARD1 may also function in vivo by assembling specific ubiquitin chains on its substrates, rather than impacting on the global polyubiquitination linkages.

### Kinase activity depends on the BRCA1 mutational status in HGSC

We next evaluated which kinases were differentially modulated and could be responsible for driving the phosphorylation changes observed between BRCA1mut and BRCA1wt tumors. Eighteen highly interconnected kinases were identified as being modulated in a BRCA1-dependent manner by different layers of regulation (Fig. 4B). In addition, some of these kinases, including CDK12, CDK7, and PRKDC, are known to be functionally related to BRCA1 through their involvement in DNA repair and transcriptional regulation^42–44^. Due to the high dynamicity and low stoichiometry of modified signaling proteins, it is possible that several other kinases, not identified in our analysis, could be involved in regulating HGSC in a BRCA1-dependent manner. In order to identify additional kinases, we used all the differentially regulated phosphorylation sites (n = 466) as readout for kinase activity and looked at the different kinase consensus sequences (Fig. 4C). Because residues that confer specificity to kinase consensus motifs are often overlapping, we next retrieved site-specific information for experimentally annotated kinase-substrate relationships from PhosphoSitePlus^29^. Significantly regulated phosphorylation sites were assigned to their upstream kinase and the relative changes in substrate phosphorylation were then used to infer kinase activity. Of the 466 differentially regulated phosphorylation sites, upstream kinases were reported in 35 sites corresponding to 33 proteins (Table S5). In order to further explore the kinase-substrate relationships we performed an analysis of those sites for which site-specific information was lacking. We used the KinomeXplorer integrated platform^45^, restraining the analysis to those kinases that we identified in our proteome, phosphoproteome and/or ubiquitinome HGSC datasets and therefore had experimental evidence. Comparison of the prediction score distributions identified CK2 and CLK groups as showing lower activity in BRCA1mut tumors (p < 0.05, Kolmogorov–Smirnov test). Protein kinase CK2 is a constitutive enzyme involved in many cellular processes including cell proliferation, apoptosis, RNA splicing and DNA repair^46,47^. BRCA1 is a known substrate of CK2 phosphorylation and mutations at specific residues can alter its interaction. However, how BRCA1 and CK2 interplay to regulate cancer-related events remains unknown. CLK kinases are involved in pre-mRNA splicing together with SRPK kinases and SR proteins through the recognition of a conserved serine-arginine enriched motif in their protein substrates. Interestingly, we observed a lower phosphorylation of proteins containing this conserved motif in BRCA1mut tumors and a reduced SRPK1 protein kinase abundance in BRCA1mut tumors. These results suggest that CLK and SRPK kinases are involved in the regulation of RNA splicing in a BRCA1-dependent manner and that BRCA1 might be regulating their transcriptional activity. The analysis of RNA sequencing data from the TCGA data set^1^ showed a trend towards reduced mRNA levels of the CK2 alpha subunit (CSNK2A), CLK and SRPK kinase members in BRCA1mut tumors, thus reinforcing the notion that BRCA1 modulates the transcriptional regulation of these kinases (Fig. 4D–F).

### PI3K/AKT/mTOR signaling, RNA splicing and DNA repair are deregulated in BRCA1 mutated HGSC tissues

**Figure 5:**
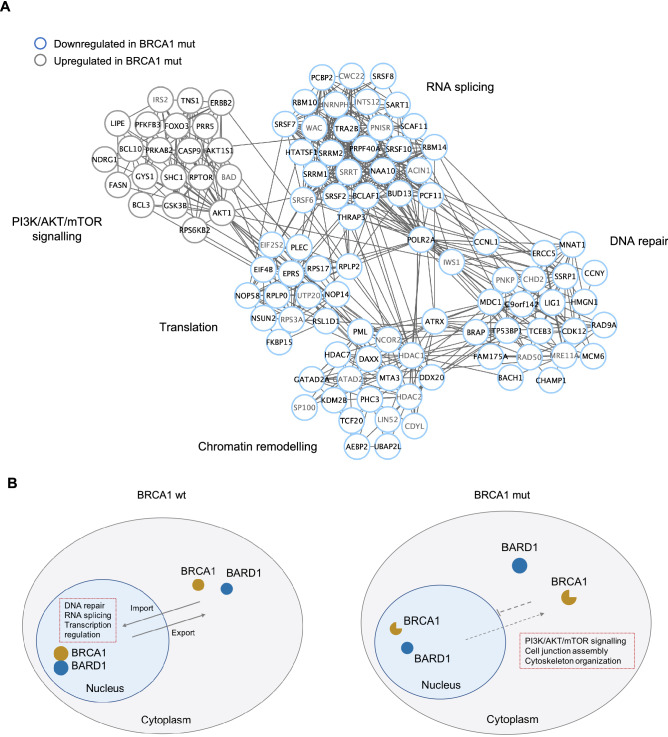
BRCA1-dependent phosphorylation signaling events and subcellular localization in ovarian cancer. **(A)** Functional network analysis of the regulated phosphorylated proteins identified in our study (nodes with black label) and their interacting proteins (nodes with grey label) (q-value < 0.05). The edges represent known protein–protein interactions based on the InWeb_IM database (interaction score > 0.9) and visualized with Cytoscape (ClusterOne plugin). RNA splicing, p < 0.001; translation, p < 0.001; DNA repair, p = 0.002; chromatin remodeling, p = 0.007; PI3K/AKT/mTOR pathway, p = 0.009 (one-tail Mann–Whitney U test). **(B)** Schematic representation of BRCA1 subcellular compartmentalization in BRCA1wt and BRCA1mut tumors. Changes in subcellular localization may be caused by changes in protein shuttling and import into the nucleus, thus reflecting in differential cellular signaling events in ovarian cancer tumors.

We next checked the signaling processes in which the differentially phosphorylated proteins (n = 345) were involved (Fig. S3A). For this we performed a clustering analysis through the generation of a protein–protein interaction network based on the InWeb_InBioMap (InWeb_IM) database^39^. Clustering analysis showed enrichment of phosphoproteins involved in RNA splicing, translation, DNA repair, chromatin remodeling and PI3K/AKT/mTOR signaling (Fig. 5A). Interestingly, RNA splicing, DNA repair and chromatin remodeling components were predominantly enriched in BRCA1wt tumors. Contrary, PI3K/AKT/mTOR signaling proteins were mostly enriched in BRCA1mut tumors (Fig. S3B,C).

Within the DNA repair cluster, we identified changes in the phosphorylation status of several BRCA1 interactors including FAM175A (*Abraxas*) at S386 and S387, MDC1 at T448 which is known to control BRCA1 localization at DNA damage sites by regulating ATM-dependent phosphorylation events, LIG1 at T195 and RAD9A at S368 and S387 which is part of the 9-1-1 complex^48–51^.

In addition to DNA repair, our analysis also captured regulation of proteins involved in RNA splicing. Of the 21 proteins belonging to the RNA splicing cluster (Fig. 5A) only BCLAF1, THRAP3 and POLR2A have been functionally related, directly or indirectly, to BRCA1 in vitro in other cellular types^52–55^. We found a reduction in phosphorylation of these three proteins together with other RNA splicing regulators and components of the splicing machinery including RBM10, SART1, SCAF11 and several SR proteins. It is quite surprising to find a reduced phosphorylation of a set of RNA splicing factors associated to BRCA1 mutations considering that globally, HGSC is associated to an increase in RNA metabolism and splicing (Fig. S2D). These findings indicate that BRCA1 might be regulating specific splicing events in HGSC, either through BCLAF1 and THRAP3, or through other splicing factors identified in our study. We also identified a close interconnection between proteins involved in RNA splicing and DNA repair suggesting BRCA1 could also be playing an important role in regulating these two processes in vivo, probably through the transcriptional regulation of specific DNA damage repair genes.

Finally, our results also showed an increase in phosphorylation events in components of the PI3K/AKT/mTOR signaling pathway. These include AKT1S1 (PRAS40) at S203 and RPTOR at S859, S863 and S877, which are both components of the mTORC1 complex, GSK3B at S9, CASP9 at S307 and ERBB2 at T1052 (Figs. 4B and 5A). This pathway is involved in cellular growth and proliferation, thus it is likely to play an important role in the survival and progression of BRCA1mut tumors as well as being a potential therapeutic target^56^. Moreover, several phosphatases showing a differential regulation (TNS2, ILKAP, MTMR1) were mainly associated to the PI3K/AKT/mTOR signaling pathway^57–59^ thus strengthening the importance of the PI3K/AKT/mTOR pathway in regulating HGSC in a BRCA1-dependent manner (Fig. 4B). Finally, twelve ubiquitin-related enzymes and DUBs were also found differentially regulated by phosphorylation and ubiquitination (Fig. 4B). These proteins were mainly involved in DNA repair, immune response and PTEN/PI3K/AKT signaling thus reinforcing the presence of a BRCA1-dependent phosphorylation and ubiquitination crosstalk in HGSC^60–63^.

### Differential subcellular compartmentalization of BRCA1 activity in HGSC patient-derived tissues

Differences in the signaling events observed between BRCA1mut and BRCA1wt tumors might reflect a subcellular compartmentalization of the expression of BRCA1-dependent proteins. Although BRCA1 is known to function primarily in the nucleus, we observed that BRCA1mut tumors showed increased protein functions taking place in the cytosol (Figs. 5B and S3D). It is thought that masking of the nuclear localization signals (NLS) of BRCA1 can impair protein shuttling between the nucleus and the cytoplasm^64^. These NLS are located in exon 11, the most common site for BRCA1 mutations in ovarian cancer^65^. The nuclear entry of BRCA1 can be regulated through its cytoplasmatic retention and the presence of particular mutations. Cytoplasmatic retention is mediated by BRAP which specifically binds to the NLS and competes for the interaction with nuclear importin receptor alpha (KPNA)^66^. Interestingly, we observed increased levels of phosphorylated BRAP and nuclear importin receptor alpha subunit 3 (KPNA3) in BRCA1 mutated HGSC. It is thought that mutations in or close to the BRCT domain of BRCA1 can have a broad impact on the overall conformation of the protein and have been associated to a shift of BRCA1 from the nucleus to the cytoplasm^67^. The consequences of BRCA1 subcellular localization, possibly due to changes in protein shuttling, has never been evidenced in human tissues and could explain the differences in cellular signaling events observed between BRCA1mut and BRCA1wt HGSC tumors (Fig. 5B). Although this observation may also be explained by BRCA1wt tumors representing a different molecular profile of HGSC compared to BRCA1mut tumors, based on the findings from our study we believe a deficient shuttling is plausible. Altogether, we show that both ubiquitination and phosphorylation are crucial in the de-regulation of HGSC in a BRCA1-dependent manner associated with a differential subcellular compartmentalization of proteins and their signaling processes.

## Discussion

Despite the importance of identifying BRCA1 mutations in the clinical setting for the management of HGSC, our understanding of the molecular phenotypes associated with BRCA1 mutations remains poor. A deeper characterization of these tumors could allow the identification of new therapeutic targets beyond PARP inhibitor therapy aimed at optimizing patient care. This study provides a comprehensive molecular understanding of HGSC tumor physiology and its dependence on BRCA1. The use of a multi-layered approach has allowed the integration of proteome, phosphorylation and ubiquitination data obtained directly from patient tissues using MS-based proteomics for the assessment of signaling processes in HGSC. Although large-scale proteomic studies are able to detect thousands of modified proteins from complex samples, such as human tissues, the analysis of post-translational modifications (PTM) remains challenging due to their inherent low stoichiometry and transient nature. In addition, patient-derived tissues carry inherent heterogenous backgrounds which can hamper the identification of biological differences. In this study we identified relevant molecular information related to the cellular physiological states of tumors and we provide relevant quantitative information of the ubiquitinome for the characterization of HGSC. We underline the importance of using biologically relevant samples obtained directly from patients rather than classical cell-line based assays, which might not always reflect the actual cellular changes occurring in vivo. Protein ubiquitination has traditionally been associated to the targeting of proteins for proteasomal degradation. However, it is now well accepted that ubiquitination also regulates other non-proteolytic cellular processes for the promotion of tumor survival and invasion^22^. Indeed, we confirmed that protein ubiquitination regulates critical proteins involved in ovarian cancer pathogenesis together with key cancer hallmarks, many of which are now being used as targets for the development of new therapies^17^. In addition, we observed changes in ubiquitin K48- and K63-linked polyubiquitination associated with HGSC pathogenesis thus providing new insights into ubiquitin dynamics in vivo. Our study also demonstrates the existence of an extensive crosstalk between phosphorylation and ubiquitination in ovarian cancer and the identification of key regulatory bridging proteins. HUWE1, for example, is an E3 ligase known to catalyze K48- and K63-linked polyubiquitination of its substrates for the regulation of cell proliferation and DNA repair^24^. Our results support the evidence that HUWE1 may be involved in ovarian cancer pathogenesis and is required for malignant transformation of ovarian epithelial cells^68^. Further studies focusing on the role of HUWE1 and other identified bridging proteins in our study will help elucidate the specific functions associated to the ubiquitination and phosphorylation crosstalk events in HGSC and the risk of malignization.

Focusing on the BRCA1-dependent cellular events in HGSC we observed they are largely dependent on PTMs (i.e. ubiquitination and phosphorylation) rather than impacting on the global proteome. This is in keeping with the function of BRCA1 as an E3 ubiquitin ligase enzyme and its central role as a core protein in regulating multiple phosphorylation-dependent signaling events. We also evidence how the BRCA1-dependent ubiquitination signaling in HGSC tissues is modulated by some of its known substrates and other BRCA1 interactors which, to date, have not been related to its E3 ligase activity. Additionally, we identified ubiquitination changes in a substantial number of proteins, previously unrelated to BRCA1, which might correspond to novel BRCA1/BARD1 substrates or interactors providing a foundation for future studies aimed at unraveling BRCA1 E3 ligase functions and their cellular effects in vivo. Both ubiquitination and phosphorylation were found to co-regulate key signaling pathways in a BRCA1-dependent manner including DNA repair, RNA splicing, transcription regulation and the PI3K/AKT/mTOR pathway. We did not only confirm the importance of DNA repair and transcriptional regulation directly in HGSC tissues*,* but also identified RNA splicing and the PI3K/AKT/mTOR pathway as playing an important role in ovarian cancer. Splicing factors and components of the splicing machinery can be targets of intracellular cascades that link changes in splicing events to DNA damage signals. Therefore, dysregulation of these splicing factors and spliceosomal components can directly or indirectly affect DNA repair and the maintenance of genome integrity. Interestingly, BCLAF1 and THRAP3 have been linked to BRCA1 in vitro, and their depletion has been shown to enhance sensitivity to DNA damaging agents^53,54^. Unveiling the relation between RNA splicing components and BRCA1 mutations will provide new insights into why these tumors have different responses to standard chemotherapy regimens and PARP inhibitors. Upregulation of the PI3K/AKT/mTOR pathway has previously been shown to be constitutively active in BRCA1 deficient breast cancer cells in vitro^69^. These findings are in line with our results in HGSC suggesting the PI3K/AKT/mTOR pathway may also be important for the survival of BRCA1-defective ovarian tumors. Several drug inhibitors aimed at targeting different components of the PI3K/AKT/mTOR pathway are currently being assessed in phase I and II clinical trials in ovarian cancer with modest results^70,71^. Our results suggest that BRCA1mut HGSC tumors might correspond to a subgroup of patients that may benefit from pharmacological inhibition of the PI3K/AKT/mTOR pathway, thus providing a rationale for further testing these drugs, particularly in the context of PARP inhibition^72^. Our analysis also revealed a subcellular compartmentalization of BRCA1-dependent signaling processes with an increase in cytoplasmatic and reduction of nuclear proteins in BRCA1mut tumors. It is reasonable to hypothesize that the impaired nuclear function of BRCA1 (e.g. DNA repair, transcriptional regulation, RNA splicing) observed in our study is due to the mislocalization of BRCA1 to the cytoplasm where it regulates alternative signaling processes in BRCA1 mutated tumors (e.g. PI3K/AKT/mTOR signaling). This might be due to impairment of the NLS located in exon 11, a common site for BRCA1 mutations. Understanding this protein shuttling and the cellular localization of BRCA1 in human tissues might shed light into the risk of malignization, cancer progression and response to treatments associated with these mutations.

Our study is limited by the small sample size and its descriptive nature. Nevertheless, we believe in its novelty as to the best of our knowledge, no studies are available neither assessing the ubiquitinome in HGSC, nor the effect of BRCA1 mutational status on the global changes occurring at the proteome, phosphorylation and ubiquitination level in these tumors and the crosstalk between both types of modifications. Our study follows a multilayered approach with the addition of valuable ubiquitination profiling of ovarian tumors as well as providing new information on proteome, phosphorylation and ubiquitination profiling according to the BRCA1 mutational status. We also used ovarian epithelium as benign controls rather than distal fallopian tube epithelium. Although a subset of HGSC originate from ovarian surface epithelium, a significant proportion of cases initiate in distal fallopian tube epithelial cells, particularly BRCA1 mutant cases. Although comparing results obtained from distal fallopian tubes would have been beneficial, we were limited by the amount of tissue available at the time of surgery. Thus, to ensure that enough sample was available and consistent for both benign and tumor tissues in order to perform our multilayered analysis, we used epithelial ovarian tissues for all benign samples.

Overall, this multi-layered proteomic study provides a comprehensive understanding of the phosphorylation and ubiquitination signaling processes that drive HGSC pathogenesis and their dependency on BRCA1. In an era of forefront proteomic technologies, the identification of distinct pathways and cancer signatures from patient-derived tissues provide a valuable resource for future mechanistic-based studies aimed at understanding distinct cancer phenotypes, provide new opportunities for therapeutic intervention and ultimately advance towards a more personalized patient care.

## Materials and methods

### Experimental model and subject details

All tissue and blood samples were collected from women undergoing surgery at Gynecological Unit at the Vall d’Hebron Hospital (Barcelona, Spain) following standardized operating procedures approved by the Institutional Ethical Board (Comité de Ética de Investigación con Medicamentos y Comision de Proyectos del Hospital Vall d’Hebron with project number PRAMI3082015). All patients provided informed consent for sample collection and the study was conducted in accordance with the ethical standards for human experimentation established in the Declaration of Helsinki. Patients selected for the study did not have a history of breast cancer or any other malignancy and had not received prior chemotherapy treatment. Ovarian tumors from chemotherapy-naïve patients with advance stage (stages III/IV) HGSC were collected at the time of primary surgery. In order to minimize the heterogeneity among tissue types, the most representative tumor section was carefully dissected prior to storage. Ovarian epithelial samples were obtained from patients undergoing risk-reducing surgery or removal of the ovaries for benign disease. In order to minimize the effect of ischemic events on the proteome and the heterogeneity among tissue cell types, all samples were immediately snap frozen and stored at − 80 °C following collection and dissection of the representative tumor cell population. All tumors and benign tissues were reviewed by two gynecological pathologists to confirm histology. Table S1 summarizes the clinicopathological characteristics of the twenty fresh frozen tissue samples.

### Blood samples and BRCA1 testing

All patients diagnosed with advanced HGSC were routinely tested for a multiplex gene panel which included BRCA1, BRCA2, RAD51C, RAD51D, BRIP1, MLH1, MSH2 and MSH6^73^. All BRCA1mut samples included in the study tested positive for BRCA1 and negative for the rest of genes in the panel. BRCA1wt samples were included when the result of the test was negative for all genes. In addition, tumor samples from these patients were tested for the presence of BRCA somatic mutations for targeted therapeutic purposes. This information was extracted from patient clinical records to ensure that all germline BRCA1wt tumors did not harvest a BRCA1 somatic mutation. Patients undergoing risk-reducing surgery were known to be BRCA1 mutation carriers prior to tissue sample collection. A priori, the five samples obtained from patients undergoing surgery for benign disease were classified as BRCA1wt. To confirm this, a BRCA gene test was performed. Blood samples were collected from each patient prior to anesthetic induction in an EDTA tube. Samples were centrifuged (15 min, RT, 1300*g*) and the cellular pellet was separated for DNA purification using the QiAmp DNA Mini kit (Qiagen cat #51304) following manufacturer instructions. Extracted DNA was sequenced for BRCA1 and BRCA2 mutations at the Vall d’Hebron Institute of Oncology (VHIO) genomics service. All five samples tested negative for BRCA1/2 mutations.

### Protein extraction from fresh frozen tissues

Tissues were homogenized in ice cold lysis buffer (1% NP40, 0,1% sodium deoxycholate, 150 mM NaCl, 1 mM EDTA and 50 mM TrisHCl, pH 7.5) using a rotor–stator homogenizer. Protease and phosphatase inhibitors were added to the lysis buffer immediately before use. Cells were incubated on ice for 30 min and then passed repeatedly ~ 5 times through a 20G needle to ensure complete lysis. After centrifugation (15 min, 4 °C, 20,000*g*) the supernatant was separated and kept on ice. Urea-based buffer (8 M Urea, 50 mM TrisHCl, 75 mM NaCl, pH 8.8) was added to the pellets and mixed well by 5 min sonication. Following centrifugation (4 °C, 15 min, 20,000*g*) the supernatant was added to the previous lysate and quantified using BCA protein assay.

### Sample preparation for proteome and phosphoproteome analysis

Samples (500 µg) were precipitated with chilled acetone (−20 °C, overnight). After centrifugation (10 min, 4 °C, 16,000*g*) and removal of the detergent-containing supernatant, pellets were resuspended in 6 M Urea with 200 mM ammonium bicarbonate (ABC). Samples were reduced with dithiothreitol (10 mM, 37 °C, 60 min) and alkylated in the dark with iodoacetamide (20 mM, 25 °C, 30 min). The resulting protein extract was first diluted to 2 M urea with 200 mM ABC for digestion with endoproteinase LysC (1:100 w:w, 37 °C, overnight, Wako, cat #129-02541) and then diluted twofold with 200 mM ABC for trypsin digestion (1:100 w:w, 37 °C, 8 h, Promega cat # V5113). After digestion, peptide mix was acidified with formic acid 5% and desalted with a MicroSpin C18 column (The Nest Group, Inc). Five micrograms were separated for analysis of the proteome. The remaining peptides were processed for phosphopeptide enrichment using the High-Select TiO_2_ Phosphopeptide Enrichment Kit (Thermo Scientific, P/N A32993) following manufacturer instructions^74^.

### Sample preparation for ubiquitinome analysis

Samples (5 mg) were precipitated with chilled acetone (−20 °C, overnight). After centrifugation (10 min, 4 °C, 16,000*g*) and removal of the detergent-containing supernatant, pellets were resuspended in 6 M Urea with 200 mM ABC. Samples were reduced with dithiothreitol (5 mM, 37 °C, 60 min) and alkylated in the dark with iodoacetamide (10 mM, 25 °C, 30 min). The resulting protein extract was first diluted to 2 M urea with 200 mM ABC for digestion with endoproteinase LysC (1:100 w:w, 37 °C, o/n, Wako, cat #129-02541) and then diluted twofold with 200 mM ABC for trypsin digestion (1:100 w:w, 37 °C, 8 h, Promega cat #V5113). After digestion, peptide mix was acidified with formic acid 0.1% and desalted with a Hypersep C18 column (Thermo Fisher P/N 60108-305). Immunoaffinity purification of K-ε-GG peptides was performed using PTMScan Ubiquitin Remnant Motif (K-ε-GG) Kit (Cell Signaling, P/N 5562S) following manufacturer’s instructions^75^. Enriched peptides were desalted using MicroSpin C18 column (The Nest Group, Inc) prior to mass spectrometric analysis.

### Chromatographic and mass spectrometric analysis

Samples were analyzed using a Orbitrap Fusion Lumos mass spectrometer (Thermo Fisher Scientific, San Jose, CA, USA) coupled to an EASY-nLC 1000 (Thermo Fisher Scientific (Proxeon), Odense, Denmark). Peptides were loaded directly onto the analytical column and were separated by reversed-phase chromatography using a 50-cm column with an inner diameter of 75 μm, packed with 2 μm C18 particles spectrometer (Thermo Scientific, San Jose, CA, USA). Chromatographic gradients started at 95% buffer A and 5% buffer B with a flow rate of 300 nl/min for 5 min and gradually increased to 22% buffer B and 78% buffer A in 79 min and then to 35% buffer B and 65% buffer A in 11 min. After each analysis, the column was washed for 10 min with 10% buffer A and 90% buffer B (Buffer A: 0.1% formic acid in water and Buffer B: 0.1% formic acid in acetonitrile). The mass spectrometer was operated in positive ionization mode with nanospray voltage set at 2.4 kV and source temperature at 275 °C. Ultramark 1621 was used for external calibration of the Fourier transform (FT) mass analyzer prior to analyses and an internal calibration was performed using the background polysiloxane ion signal at *m/z* 445.12003. The acquisition was performed in data-dependent acquisition (DDA) mode and full MS scans with 1 microscans at resolution of 120,000 were used over a mass range of *m/z* 350–1500 with detection in the Orbitrap mass analyzer. Auto gain control (AGC) was set to 1E5 and charge state filtering disqualifying singly charged peptides was activated. In each cycle of DDA analysis, following each survey scan, the most intense ions above a threshold ion count of 10,000 were selected for fragmentation. The number of selected precursor ions for fragmentation was determined by the “Top Speed” acquisition algorithm and a dynamic exclusion of 60 s. Fragment ion spectra were produced via high-energy collision dissociation (HCD) at normalized collision energy of 28% and they were acquired in the ion trap mass analyzer. AGC was set to 1E4, and an isolation window of 1.6 *m/z* and a maximum injection time of 200 ms were used. All data were acquired with Xcalibur software v.4.1.31.9. Digested bovine serum albumin (New England BioLabs cat #P8108S) was analyzed between each sample to avoid sample carryover and to ensure stability of the instrument. QCloud was used to control instrument longitudinal performance during the data set acquisition^76^.

### Peptide identification and quantification

Raw data were analyzed with MaxQuant software v.1.6.0.16 using the Andromeda search engine^77,78^. Data were searched against a Swiss-Prot human database (as in April 2018, 20,341 entries) plus a list of common contaminants (148 entries)^79^. Trypsin was chosen as enzyme and up to two missed cleavages were allowed. For peptide identification a mass tolerance of 4.5 ppm on precursor masses and 0.5 Da on fragment ions was set. Only peptides with an Andromeda score > 40 were included. Oxidation of methionine, N-terminal protein acetylation, phosphorylation of serine, threonine and tyrosine, and ubiquitination of lysine were used as variable modifications. Cysteine carbamidomethylation was set as a fixed modification. False discovery rate (FDR) in peptide identification was set to a maximum of 5%. At least one unique peptide was considered per protein for it to be identified and reported. The rest of parameters were set as default^80^. Peptides were quantified with extracted ion chromatogram areas using the embedded algorithm from MaxQuant. Protein abundances were estimated from peptide areas using the LFQ algorithm^81^.

### Data analysis

Data analysis was performed with Perseus software v.1.6.2.1^82^. For all data comparisons 60% of valid values were considered and log2-transformed. For the analysis of phosphorylated and ubiquitinated peptides, data were centered to the median and only peptides with a site-localization probability of at least 0.75 (class I sites) were included in the analysis. Changes in protein or peptide abundance between groups were compared using two-sided *t* test analysis followed by correction for multiple testing^83^. Changes were considered significant with a q-value below 0.05. Hierarchical clustering was performed using Pearson correlation and average linkage clustering. Heat maps were performed using Instant Clue software v.0.5.2^84^. Gene Ontology terms and Reactome pathways were analyzed with WebGestalt using the default settings^85^. Overrepresented terms and pathways were considered with a Benjamini–Hochberg corrected p-value less than 0.05. In order to reduce the redundancy of enriched gene sets, affinity propagation clustering was performed using the R package apcluster^86^.

### Protein–protein interaction networks

Protein interaction networks of the regulated proteins were obtained using the STRING database v.11^87^ and visualized on Cytoscape v.3.7.1^88^. Clusters represented in the protein interaction networks were performed using the ClusterONE cytoscape plug-in^89^. The BioGrid v.3.5 and InWeb_InBioMap (InWeb_IM) v.2016_09_12 databases were searched to obtain the list of BRCA1 interacting proteins^39,40^. The InWeb_IM database probabilistic score model was also used to infer protein interactions from our set of regulated proteins using a minimum filter score of 0.9. Representation and visualization of integrated Reactome pathways were performed using the Reactome Knowledgebase graph database and the Cytoscape ReactomeFI plug-in^90,91^.

### Kinase prediction analysis

For the identification of kinase motifs, the sequence windows of the regulated phosphorylation sites were analyzed with pLOGO^92^. The human protein sequence database was set as background and a p-value of 0.05 as cut-off. Kinase-substrate relationships were obtained from the PhosphositePlus database^29^. Prediction of unknown kinase-substrate relationships was performed with KinomeXplorer^45^. All the differentially expressed phosphosites were used as NeworKIN queries and the prediction scores for the likely kinase that phosphorylated each site were retrieved. For sites with no predicted kinases or those with only one kinase group member the NetPhorest score was also calculated. For each kinase the distribution of prediction scores between upregulated and downregulated substrate groups were compared using two-sided Kolgamorov-Smirnov test. Those kinases with a p-value < 0.05 were considered to have significant predictive activities. One-sided Kolgamorov-Smirnov test was used to assess if the kinases were predicted to be more or less active between groups.

### TCGA datasets

The ovarian serous cystadenocarcinoma TCGA dataset was reviewed to obtain information on the genomic and transcriptomic data^1^. RNA-seq level-3 data was downloaded from the Broad Institute Firehouse pipeline (307 cases) (http://gdac.broadinstitute.org/). Information on the genetic mutations for each case was downloaded from cBioPortal (Cerami et al., 2012). Cases were filtered for those having mutations in BRCA1 (26 cases). The rest were manually checked to exclude those with BRCA2 mutations or any of the other six genes included in our gene panel test. The remaining cases were classified as BRCAwt. This left us with a total of 288 cases for RNA-seq analysis. For data visualization, mRNA expression values were z-score normalized to standard deviations from the mean. At least 60% of valid values in each group were considered for analysis. Graphs were plotted on GraphPad Prism Software for visual representation and statistical analysis using two-sided Mann–Whitney U test. A p-value cut-off of 0.05 was considered significant.

## Supplementary Information

Supplementary Table S1.

Supplementary Table S2.

Supplementary Table S3.

Supplementary Table S4.

Supplementary Table S5.

Supplementary Figures.

## Data Availability

The mass spectrometry data have been deposited to the PRIDE repository^93^ with the dataset identifier PXD020271.
