# Supplementary material for: BRCA1 mutations in high-grade serous ovarian cancer are associated with proteomic changes in DNA repair, splicing, transcription regulation and signaling
Source: Sci Rep. 2022 Mar 15;12:4445. doi: 10.1038/s41598-022-08461-0 (PMC8924168; doi:10.1038/s41598-022-08461-0)
Supplement: Supplementary file 6 — Supplementary Figures. [file 41598_2022_8461_MOESM6_ESM.pdf]

SUPPLEMENTAL FIGURES

Figure S1: Proteomic characterization of twenty ovarian tissues.

(A) Distribution of the peptide mass error and **(B)** intensity range. **(C)** Distribution of phosphorylated peptides with one (single), two (double) or more (> double) phosphorylated sites. **(D)** Distribution of serine (pSer), threonine (pThr) and tyrosine (pTyr) phosphorylation sites. **(E)** Distribution of ubiquitinated peptides as in (D). **(F)** Venn diagram showing the overlap between the proteome and the **(G)** phosphorylated proteins identified in this study and in two reference studies by (Zhang et al., 2016; Francavilla et al., 2017).

Figure S2: Related to figure 1. Proteome and phosphoproteome analysis in ovarian cancer and benign tissues.

(A) Heatmap of the Pearson's correlation of the proteome, **(B)** phosphoproteome and **(C)** ubiquitinome data show good overall reproducibility between cancer and benign samples [84]. **(D)** GO Biological Processes (GOBP) term enrichment analysis of the most significantly regulated proteins from the proteome dataset (q-value < 0.01 and log2 fold change > 2). The analysis included 527 proteins. **(E)** GOBP enrichment analysis of the 473 proteins with significant changes in their phosphorylated status (q-value < 0.01).

Figure S3: BRCA1-dependent phosphorylation and ubiquitination signaling events in ovarian cancer.

(A) Hierarchical clustering of the differentially expressed phosphorylated sites between BRCA1mut and BRCA1wt high-grade serous cancer (HGSC) tissues. Protein intensity is presented in logarithmic scale. Low intensity proteins are depicted and blue and high intensity proteins in red [82]. **(B)** GO Biological Processes and **(C)** Reactome enrichment analysis of the differentially regulated phosphorylated proteins (q-value < 0.05). Colored bars indicate downregulation (blue) and upregulation (yellow) in BRCA1mut tumors. **(D)** Network showing the different signaling pathways modulated by all three layers of regulation (q-value < 0.05): proteome (red), phosphorylation (green), ubiquitination (blue) and both post-translational modifications (purple). Components of each pathway were extracted from the Reactome database and visualized with Cytoscape.

Figure S1

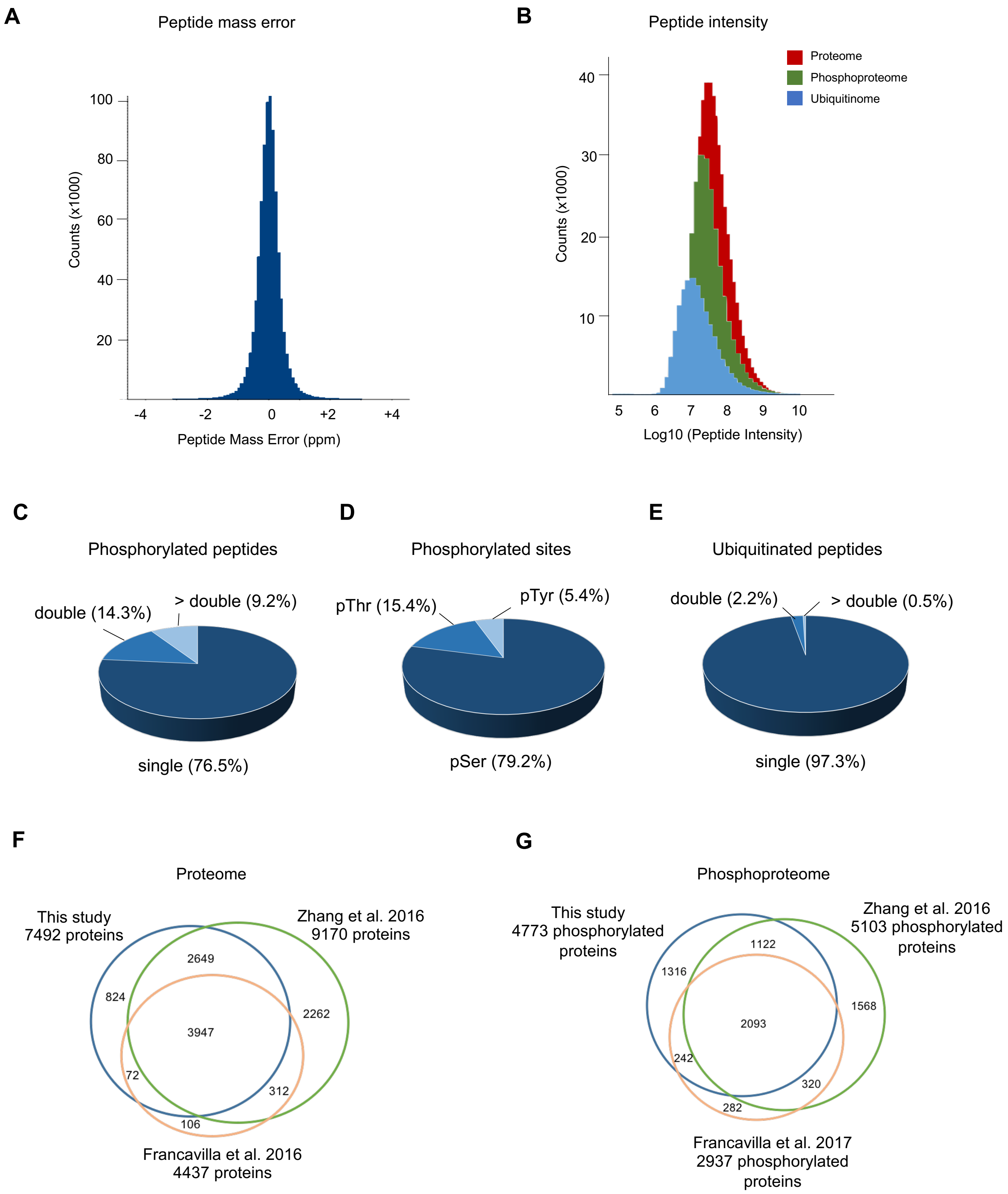

A
Peptide mass error
Counts (x1000)
100
80
60
40
20
-4
-2
0
+2
+4
Peptide Mass Error (ppm)
B
Peptide intensity
Proteome
Phosphoproteome
Ubiquitinome
Counts (x1000)
40
30
20
10
5
6
7
8
9
10
Log10 (Peptide Intensity)
C
Phosphorylated peptides
double (14.3%)
> double (9.2%)
single (76.5%)
D
Phosphorylated sites
pThr (15.4%)
pTyr (5.4%)
pSer (79.2%)
E
Ubiquitinated peptides
double (2.2%)
> double (0.5%)
single (97.3%)
F
Proteome
This study
7492 proteins
Zhang et al. 2016
9170 proteins
2649
824
2262
3947
72
312
106
Francavilla et al. 2016
4437 proteins
G
Phosphoproteome
This study
4773 phosphorylated
proteins
Zhang et al. 2016
5103 phosphorylated
proteins
1122
1316
1568
2093
242
320
282
Francavilla et al. 2017
2937 phosphorylated
proteins

Figure S2

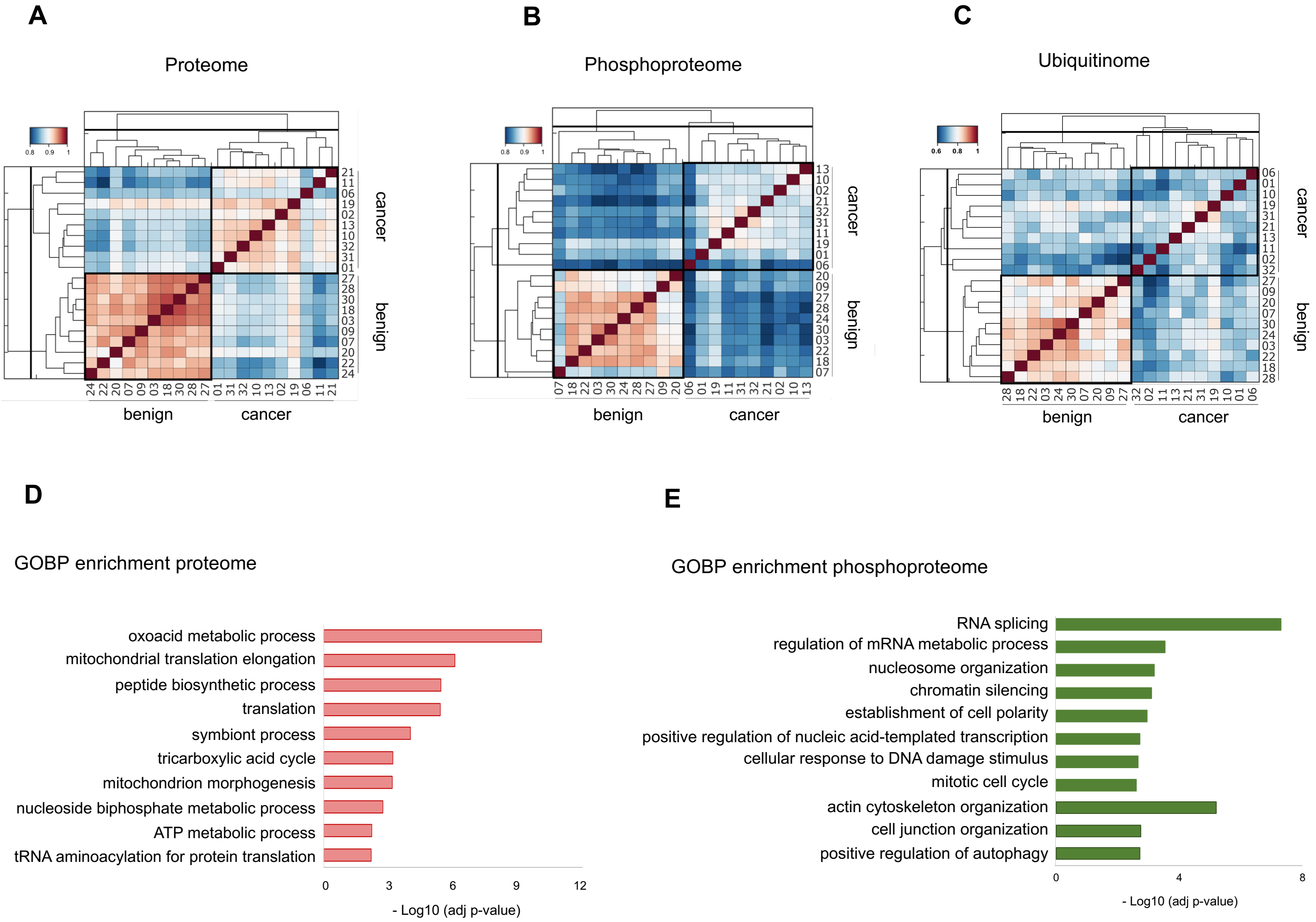
A
Proteome
0.8 0.9 1
cancer
benign
benign
cancer
B
Phosphoproteome
0.8 0.9 1
cancer
benign
benign
cancer
C
Ubiquitinome
0.6 0.8 1
cancer
benign
benign
cancer
D
GOBP enrichment proteome
oxoacid metabolic process
mitochondrial translation elongation
peptide biosynthetic process
translation
symbiont process
tricarboxylic acid cycle
mitochondrion morphogenesis
nucleoside biphosphate metabolic process
ATP metabolic process
tRNA aminoacylation for protein translation
0 3 6 9 12
- Log10 (adj p-value)
E
GOBP enrichment phosphoproteome
RNA splicing
regulation of mRNA metabolic process
nucleosome organization
chromatin silencing
establishment of cell polarity
positive regulation of nucleic acid-templated transcription
cellular response to DNA damage stimulus
mitotic cell cycle
actin cytoskeleton organization
cell junction organization
positive regulation of autophagy
0 4 8
- Log10 (adj p-value)

Figure S3

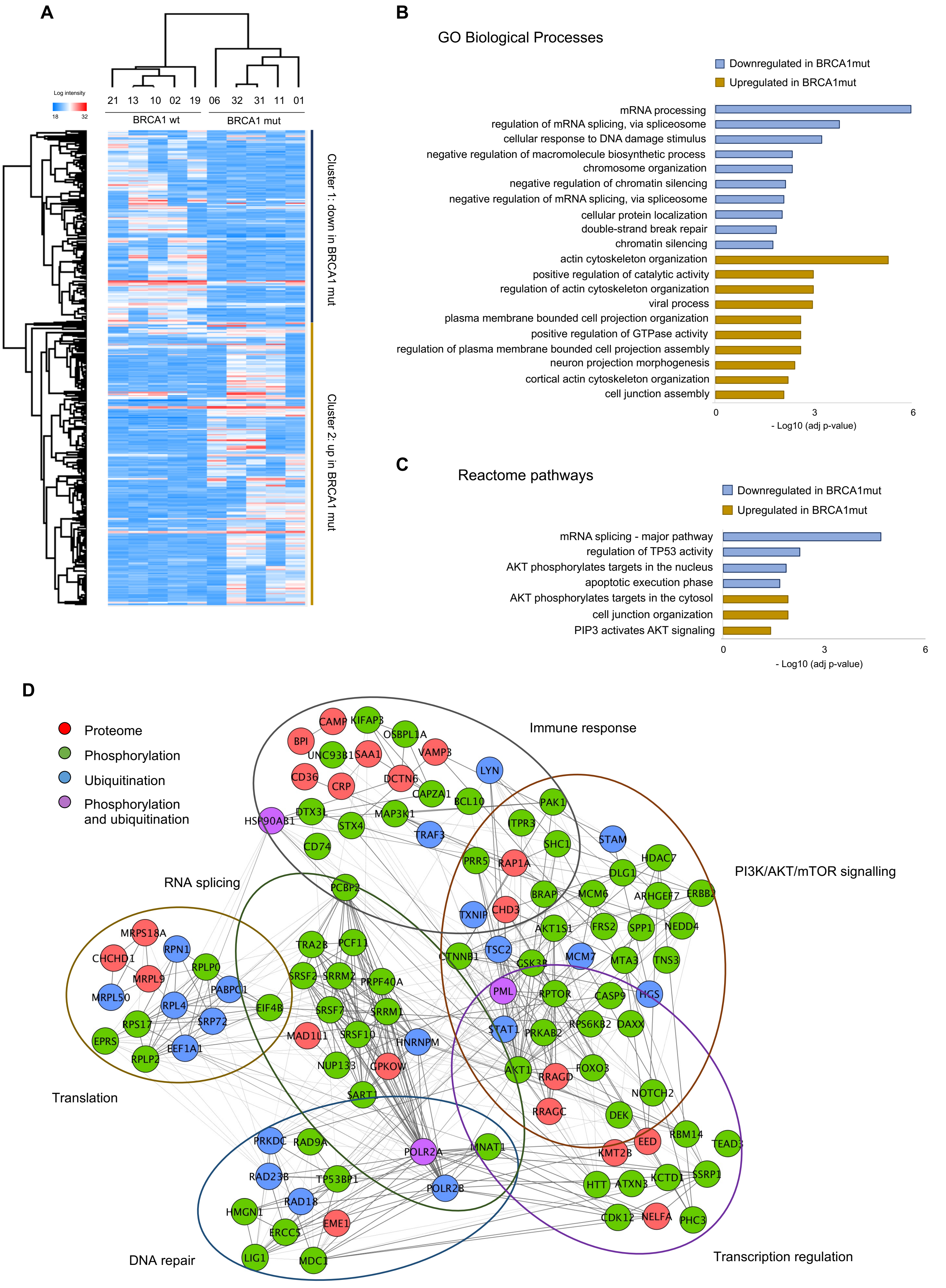
A
Log intensity
18
32
21 13 10 02 19 06 32 31 11 01
BRCA1 wt
BRCA1 mut
Cluster 1: down in BRCA1 mut
Cluster 2: up in BRCA1 mut
B
GO Biological Processes
Downregulated in BRCA1mut
Upregulated in BRCA1mut
mRNA processing
regulation of mRNA splicing, via spliceosome
cellular response to DNA damage stimulus
negative regulation of macromolecule biosynthetic process
chromosome organization
negative regulation of chromatin silencing
negative regulation of mRNA splicing, via spliceosome
cellular protein localization
double-strand break repair
chromatin silencing
actin cytoskeleton organization
positive regulation of catalytic activity
regulation of actin cytoskeleton organization
viral process
plasma membrane bounded cell projection organization
positive regulation of GTPase activity
regulation of plasma membrane bounded cell projection assembly
neuron projection morphogenesis
cortical actin cytoskeleton organization
cell junction assembly
0 3 6
- Log10 (adj p-value)
C
Reactome pathways
Downregulated in BRCA1mut
Upregulated in BRCA1mut
mRNA splicing - major pathway
regulation of TP53 activity
AKT phosphorylates targets in the nucleus
apoptotic execution phase
AKT phosphorylates targets in the cytosol
cell junction organization
PIP3 activates AKT signaling
0 3 6
- Log10 (adj p-value)
D
Proteome
Phosphorylation
Ubiquitination
Phosphorylation and ubiquitination
Immune response
PI3K/AKT/mTOR signalling
RNA splicing
Translation
DNA repair
Transcription regulation
CAMP KIFAP3 BPI OSBPL1A UNC93B1 SAA1 VAMP3 CD36 CRP DCTN6 LYN CAPZA1 BCL10 PAK1 HSP90AB1 DTX3L MAP3K1 STX4 ITPR3 TRAF3 CD74 SHC1 STAM PRR5 RAP1A HDAC7 DLG1 BRAP MCM6 ARHGEF7 ERBB2 TXNIP CHD3 PCBP2 AKT1S1 FRS2 SPP1 NEDD4 MRPS18A RPN1 CHCHD1 RPLP0 MRPL9 MRPL50 PABPC1 RPL4 SRP72 RPS17 EPRS EEF1A1 RPLP2 EIF4B TRA2B PCF11 SRSF2 SRRM2 PRPF40A SRSF7 SRRM1 MAD1L1 SRSF10 HNRNPM NUP133 GPKOW SART1 CTNNB1 TSC2 GSK3B MCM7 MTA3 TNS3 PML RPTOR CASP9 HGS STAT1 PRKAB2 RPS6KB2 DAXX AKT1 RRAGD FOXO3 NOTCH2 RRAGC DEK EED RBM14 TEAD3 KMT2B PRKDC RAD9A POLR2A NMNAT1 RAD23B TP53BP1 POLR2B HTT ATXN3 KCTD1 SSRP1 RAD18 HMGN1 EME1 ERCC5 CDK12 NELFA PHC3 LIG1 MDC1
